# Clinical performance evaluation of a sensitive, rapid low-throughput test for *KRAS* mutation analysis using formalin-fixed, paraffin-embedded tissue samples

**DOI:** 10.1186/s12885-017-3112-0

**Published:** 2017-02-16

**Authors:** Christine Weyn, Sofie Van Raemdonck, Robina Dendooven, Vincent Maes, Karen Zwaenepoel, Suzan Lambin, Patrick Pauwels

**Affiliations:** 1 Pathology Department, University Hospital Antwerp, Wilrijkstraat 10, 2650 Edegem, Belgium; 2 Centre for Oncological Research (CORE), University of Antwerp, Universiteitsplein 1, 2610 Wilrijk, Belgium

**Keywords:** KRAS, Metastatic colorectal carcinoma, FFPE, Mutation analysis, Idylla

## Abstract

**Background:**

Testing for *KRAS* mutations in metastatic colorectal cancer (mCRC) on formalin-fixed, paraffin embedded (FFPE) tumor tissue has become standard of care. Different molecular methods exist to determine hotspot *KRAS* mutations in exon 2, 3 and 4, but testing is often limited by the sensitivity and the speed of analysis. The aim of this retrospective study was to establish the clinical performance of the Idylla™ KRAS Mutation Test on FFPE tumor samples of patients with mCRC.

**Methods:**

*KRAS* mutation analysis was performed using the *therascreen KRAS* on the RotorGene Q platform (CE-IVD; Qiagen) and results were subsequently compared to the Idylla™ KRAS Mutation Test. Discordant result testing was performed with massive parallel sequencing or alternative routine approaches.

**Results:**

Data from 182 samples were used to show that the overall agreement between the two methods for mutation characterization was 96.7% [95%CI: 93.0%-98.5%]. Six out of 182 samples (3.3%) showed true discordant results.

**Conclusion:**

The Idylla™ KRAS Mutation Test allows for a fast and reliable analysis of FFPE samples with a turnaround-time of two hours without the need of molecular infrastructure or expertise in order to guide the personalized treatment of colorectal cancer patients.

## Background

Colorectal cancer (CRC) is the fourth most common cause worldwide of cancer and counts for approximately 10% of cancer related mortalities in western countries [1, 2].

Treatment options for metastatic CRC include targeted therapies with monoclonal antibodies (mAbs), namely cetuximab (Erbitux, Merk KgaA, Darmstadt, Germany) and panitumumab (Vectibix, Amgen Thousand Oaks, CA, United States) [3]. These molecules both target the extracellular domain of the epidermal growth factor receptor (EGFR) protein and compete with ligands, leading to the blocking of ligand induced intracellular signal transmission. Both cetuximab and panitumumab have been shown to improve survival in mCRC patients, both as monotherapy as well as in combination with conventional chemotherapies [4–7]. However, mCRC patients whose tumors harbor mutations in the rat sarcoma viral oncogene homolog (*RAS*) gene family, including the kirsten *RAS* (*KRAS*) and neuroblastoma *RAS* (*NRAS*) proto-oncogenes, do not benefit from therapy with these mAbs [6, 8, 9].

This is due to the constitutive activation of the mutated proteins, independently of ligand binding. As a consequence, testing of the RAS mutation status in mCRC patients functions as predictive marker to guide therapy with anti EGFR-antibodies [10–12].

Based on the pooled analysis of the *RAS* status of over 3000 patients, the overall prevalence of *RAS* mutations was calculated as being 55.9%, with the majority of these mutations being present in *KRAS* exon 2 (42.6%). Mutations in *KRAS* exon 3 (3.8%), *KRAS* exon 4 (6.2%) and *NRAS* exon 2 (2.9%), *NRAS* exon 3 (4.2%) and *NRAS* exon 4 (0.3%) were shown to be less prevent, but still account for over 15% of all *RAS* mutations in the mCRC setting [13]. Hence, extended RAS testing of tumor tissue (primary or metastatic) beyond *KRAS* exon 2 is now recommended both by the European Society of Medical Oncology (ESMO) and by the National Comprehensive Cancer Network (NCCN) [3, 6, 8].

Various molecular techniques exist to detect KRAS mutations, each with their advantages and disadvantages such as differences in cost, test duration, sensitivity, specificity, reproducibility, capacity to quantify the mutated alleles and ability to detect new mutations [14–16]. Only two methods currently available to test for *KRAS* mutations in FFPE samples are approved by the Food and Drug Administration, namely the *therascreen KRAS* RGQ PCR Kit (Qiagen Manchester Ltd, Manchester, UK) and the cobas® *KRAS* Mutation Test (Roche, Branchburg, NJ, USA) [17]. Briefly, both methods require tissue deparaffinization, extraction of genomic DNA from formalin-fixed, paraffin-embedded (FFPE) tissue, DNA quantitation and followed by quantitative polymerase-chain reaction (qPCR) on specific instruments. Extensive data-analysis is not required. The *therascreen KRAS* RGQ PCR Kit allows the detection of seven mutations in codons 12 and 13, while the cobas® KRAS Mutation Test additionally detects mutations in codon 61. This latter method however does not allow full characterization of the individual mutations. Both methods are equally labor-intensive and require a turnaround-time of 3-4 h. Also, both techniques preferentially use pooling of several samples in view of the optimal use of the kit, often leading to a more prolonged turnaround-time.

The Idylla™ KRAS Mutation Test (Biocartis, Mechelen, Belgium) is CE-IVD labeled and allows characterization of 21 hot-spot *KRAS* mutations in exons 2, 3 and 4, namely G12D, G12A, G12C, G13D, G12V, G12S, G12R, A59T/E/G, Q61H, Q61K, Q61R/L, K117N and A146P/T/V. Furthermore, this test does not require separate deparaffinization, DNA quantification and genomic DNA isolation, since all reactions for deparaffinization, DNA extraction and PCR are fully automated and performed in a single-use cartridge. This study aimed at comparing the clinical performance of the Idylla™ KRAS Mutation Test to the *therascreen* KRAS RGQ PCR Kit for 182 valid results obtained from mCRC FFPE samples. Comparison includes the overall percentage agreement, percent positive agreement and percent negative agreement, defined as percentages of valid Idylla™ results in agreement with or different from the comparator method. Discordant samples were confirmed with alternative routine approaches.

## Methods

### Tissue specimens

This study was approved by the Ethical committee of the University Hospital Antwerp (UZA) and includes FFPE tumor samples from 230 patients with mCRC that were referred for *KRAS* mutation analysis at our institute (UZA) between 2010 and 2015. Additionally, 22 commercial samples were provided by Biocartis to UZA, bringing the total number of samples to 252.

Of these samples, 104 (41.3%) had been collected less than 1 year before testing, 53 (21.0%) between 1 and 2 years, 45 (17.86%) between 2 and 3 years, 36 (14.3%) between 3 and 4 years and 14 (5.6%) between 4 and 5 years. Older samples could not be tested due to restrictions imposed by the institutional review board.

The study was conducted at UZA where the Idylla™ as well as the *therascreen* KRAS RGQ PCR reference test were performed. From the 252 eligible FFPE samples analyzed, 77 (30.56%) were metastatic tissue samples and 171 samples (67.86%) were derived from the primary tumor. For four samples, the tumor origin was unknown.

Based on histological assessment of H&E staining, consecutive slides of the samples were enriched by manual macrodissection to reach a tumor content of at least 25%. These samples were subsequently tested with the Idylla™ KRAS Mutation Test (IUO) or with the reference test. The influence of necrotic tissue on the results was evaluated.

### Mutation detection by the Idylla™ molecular diagnostic system

Ready-to-use Idylla™ KRAS Mutation Test cartridges (IUO), allowing the detection of mutations in codons 12, 13, 59, 61, 117 and 146 of the *KRAS* gene, were used (G12D, G12A, G12C, G12V, G12S, G12R, G13D, A59T/E/G, Q61H/Q61H, Q61K/Q61K, Q61R/L, K117N/K117N and A146P/T/V) were provided by the company (Biocartis, Mechelen, Belgium). These cartridges contain the necessary reagents to perform sample preparation, real-time PCR amplification and detection, starting from insertion of FFPE tissue into the cartridge. Briefly, the process steps in the test are the FFPE liquefaction and cell lysis followed by real-time PCR using allele specific primers. Amplification of a *KRAS* sequence in intron4/exon5, serving as a sample processing control, is included in each run. The presence of a mutant genotype is determined by calculating the difference between the *KRAS* Sample Processing Control Cq and the Cq obtained for the *KRAS* mutant signal(s). In case of multiple mutations, only the dominantly detected mutation (lowest ΔCq value) is currently reported.

Idylla™ analyses were performed according to the manufacturer’s recommendations for investigational use. Briefly, a tumor area of at least 50 mm^2^ (for 5 μm slices) per sample was transferred into the cartridges. The time between preparation of the slide(s) and the actual testing should not exceed 60 days. A tumor tissue content of at least 25% was obtained, if needed after macrodissection, allowing the detection of mutations present with an allelic frequency between 1% for G12R and ~15% for A146V/T/P in this investigational phase of the assay. The performance characteristics of the CE-IVD Idylla™ KRAS Mutation Test have been extended in the meantime for mutations with a low prevalence, meaning that all mutations down to an allele frequency of 5% were shown to be detectable. This implies that the instructions for use state a 10% tumor tissue content (TTC) requirement from July 2016 on. Repeat testing was performed once, whenever an invalid KRAS result was obtained. Invalid results may be caused by a variety of reasons including presence of inhibitors in the sample, insufficient amplifiable DNA present in the sample, incorrect placement of a sample in a cartridge, or sample volume out or range. In addition, incorrectly stored cartridges, cartridges used that exceeded their in-use period after removal from the pouch, or cartridge malfunctioning were reported as possible reasons for invalid results.

### Limit of detection

The Limit of Detection (LOD) is defined as the lowest KRAS mutation copy number consistently detected in ≥ 95% of the cases (with 95% confidence) at an allelic frequency of 5%.

Four clinical *KRAS* mutation positive FFPE specimens with 5-10% tumor cell content were included to verify the LOD. Specimens with a previously determined G12D, G12V and G12C mutation could be collected.

### Mutation detection using the *therascreen* RGQ PCR *KRAS* Kit

After deparaffinization with xylene, genomic DNA from mCRC samples was manually extracted from 5 μm slides using the QIAamp DNA FFPE Tissue Kit according to the manufacturer’s recommendation. Samples with a tumor content of at least 20% were used with a total minimal tumor area of 4 mm^2^, using one or more consecutive sections. Total amplifiable DNA was first assessed using qPCR using an internal control per sample. Samples with Cq values between 21.92 and 32.00 were considered as valid and suitable for subsequent KRAS analysis. In the event DNA was too concentrated (Cq < 21.92), the sample was diluted and re-tested. Samples with a Cq > 32 were excluded from further analysis. KRAS mutation analysis was then performed for valid samples in 8 different PCR reactions: 7 mutation reactions and 1 control reaction. The PCR run and data analysis were performed according to manufacturer’s instructions. Repeat testing was performed once, whenever an invalid KRAS result was obtained. Invalid KRAS results are due to failure of internal, negative or positive controls as stated by the manufacturer.

### Discordant testing

Targeted sequencing of discordant samples was performed using the SOMATIC1 MASTR v2 Kit (Multiplicom; Niel, BE). This kit specifically amplifies full coding regions of *KRAS*, *NRAS* and *BRAF* with short amplicons (168-255 bp). Since characterization of variants in the full coding region of the *BRAF* gene is not required, only a single-plex PCR was performed amplifying full exons of *KRAS* and *NRAS* and only exon 15 of *BRAF*, as specified by the manufacturer. Briefly, DNA quality of samples was first assessed using the QC plex, according to the instructions for use. Only samples with a DQC of > 0.12 were considered suitable for further analysis. Samples were subsequently amplified using 2-5 μl DNA (8-20 ng). The library quantification was carried out using the Qubit DNA HS Kit (Life Technologies). For sequencing on the MiSeq Illumina platform, the 600v3 sequencing reagent kit was used. Data analysis was performed with SeqNext v.4.2.1 (JSI Medical Systems, Ettenheim, Germany). Analysis was performed for samples reaching the 1000x coverage at the genomic positions of the hotspot mutations covered by the Idylla™ and *therascreen* tests.

Alternatively, Sanger sequencing was used whenever MPS was not successful in mutation detection. First, PCR was performed using the following primers: 5’- GTAAAACGACGGCCAGGTGTGACATGTTCTAATATAG-3’ (Forward) and 5’- TTGGATCATATTCGTCCACAA-3’ (Reverse) for KRAS exon 2, 5’- GTAAAACGACGGCCAGCCAGACTGTGTTTCTCCCTTCTCAGG -3’ (Forward) and 5’- AGAAAGCCCTCCCCAGTCCTCA-3’ (Reverse) for KRAS exon 3, 5’- GTAAAACGACGGCCAGTCAGATCTGTATTTATTTCAGTGTTACTTACCT-3’ (Forward) and 5’- CAGGAAACAGCTATGACCGACTCTGAAGATGTACCTATGGTCCTA-3’ (Reverse) for KRAS exon 4 (K117N) and 5’-GTAAAACGACGGCCAGTAATGACATAACAGTTATGATTTTGCAGAAAA-3’ (Forward) and 5’-CAGGAAACAGCTATGACCCAGGCTCAGGACTTAGCAAGAAG-3’ (Reverse) for KRAS exon 4 (A146/VT/P). In this reaction, after an initial denaturation step at 95 °C during 120 s, the PCR mixture was subjected to 45 rounds of amplification consisting of a 30 s denaturation at 94 °C, a 30 s annealing at 64 °C and a 30 s elongation at 72 °C. Sanger sequencing was performed using a universal M13 tag (5’-GTAAAACGACGGCCAG-3’) on an ABI3130 Instrument. Analysis was performed with SeqPatient software (JSI Medical Systems, Ettenheim, Germany).

### Diagnostic performance calculations

Overall agreement (% total agreement), negative and positive agreement was estimated together with a 95% two-sided confidence interval based on Wilson’s score method [18] at the dichotomous level, “mutation detected” versus “no mutation detected”. Percentage overall agreement is defined as the proportion of concordant results against the sum of concordant and discordant results.

Positive agreement is defined as the proportion of valid tests resulting in the detection of the mutation that are in concordance between the Idylla™ system and the comparator method against the number of all mutations detected by the comparator system.

Negative agreement is defined as the proportion of concordant tests without the mutation against the number of all comparator tests without mutation.

The statistical comparison of invalid Idylla™ KRAS Mutation Test Results for each sample collection time interval was performed with the Chi squared test [19].

## Results

### Study population and overall performance of the Idylla™ system

The Idylla™ system is a quick, on-demand system that allows fast analysis of hot spot *KRAS* mutations in exon 2, 3 and 4 starting from 50 mm^2^ tissue sections with minimum 25% tumor content in order to reach an LOD ranging between 1% ~ 15% depending on the mutation. Macrodissection was performed in 97 samples (38.5%). There was no significant difference in percentage invalid results between macro- and non-macro-dissected samples. Eight samples with a 1-10% tumor content could not be macro-dissected, eg. due to small tissue size or spread-out tumor cells, and were tested as such. In 2 out of these samples, a *KRAS* mutation was detected, which was also true for the *therascreen* comparator test. Also, 30 out of 31 samples not reaching the 50 mm^2^ cut-off tumor tissue area yielded a successful result.

It was not always possible to maintain the maximum delay of 60 days between the date of sectioning and the Idylla™ KRAS Mutation Test due to the large amount of samples tested. Overall, 60 samples (23.8%) were tested within 60 days and 192 samples (76.2%) after 60 days. There was no correlation between the number of invalid results and the overdue time (data not shown).

We investigated the possibility that a statistically significant association was present between the age of the samples, defined as the time between the collection and testing date, namely <1 year, 1–2 years, 2–3 years, 3–4 years, 4–5 years, and the number of invalid results. Overall, 7 samples turned out to be invalid, amongst which 1 sample was part of category < 1 year and 6 samples belonged to the category 4–5 years. A statistical difference was noted (*p* < 0.05) between this last category and all other categories.

In addition, the potential effect of necrotic tissue on the results was evaluated. The number of valid results did not correlate with a necrotic category, namely <10%, 11-25%, 26-50% (data not shown).

### Verification of the limit of detection of the Idylla^TM^ KRAS Mutation Test

The LOD of the Idylla™ KRAS Mutation Test was verified using clinical FFPE samples containing a low TTC (5–10%) and harboring a previously determined *KRAS* mutation. Results are shown in Table 1. The percentage agreement was 100%.

**Table 1:** Verification of LOD with clinical samples

	MPS	Idylla™ KRAS Mutation Test
16S106	G12C (10% TTC: 31%VAF)	G12C
16K11	G12C (5-10% TTC: 33% VAF)	G12C
16O1048	G12V (10% TTC; 3% VAF)	G12V
16EM117	G12D (10% TTC, 18%VAF)	G12D

*TTC* tumor tissue content, *VAF* variant allele frequency

### Comparison of KRAS specifications

The percentage overall agreement of KRAS mutation analysis took into account that the number of *KRAS* mutations detected by the *therascreen* and Idylla™ method is not completely equal. More specifically, both methods detect identical mutations in exon 2, but the Idylla™ platform additionally detects hotspot mutations in exon 3 and 4. Since the *therascreen* kit is the only FDA approved *KRAS* mutation analysis platform allowing comparison at the mutation-specific level, this was the comparator method of choice.

### KRAS mutation analysis using the *therascreen* reference method

The *therascreen* KRAS RGQ kit first required a quality check by qPCR to determine eligibility for subsequent KRAS analysis. Out of the 252 samples that fulfilled the criteria of the kit, 227 samples generated a QC result within the acceptable Cq range (90.1%). Only these 227 samples were further tested for KRAS mutation analysis and a valid result was subsequently generated for 195 samples. Thirty two samples had a non-reportable invalid result, due to test or system alerts. Hence, the overall success rate was 77.4% (195/252) with 86% valid results (195/227) and 14% invalid results (32/227). Overall, KRAS mutations in exon 2 were detected in 83 samples (42.6%).

### Comparison *therascreen* versus Idylla™ KRAS Mutation Test at dichotomous Mutation Detected level

There were 252 eligible samples of which 195 and 245 generated a valid result with *therascreen* or Idylla™, respectively. Overall, 194 samples generated a valid result with both methods. Since the *therascreen* KRAS test only detects 7 out of the 21 mutations detected by the Idylla™ KRAS Mutations Test, discordant results are obtained whenever one of the 14 other mutations (A59E, A59G, A59T, Q61K, Q61K, Q61L, Q61R, Q61H, Q61H, K117N, K117N, A146P, A146T, A146V) was detected by the Idylla™ KRAS Mutation Test. In this study there were 13 discordant-by-design mutations detected by Idylla™, of which one had an invalid result with *therascreen*. This means that overall there were 12 mutations discordant by design amongst the valid samples, which were not further taken into account for statistical analyses. Table 2 shows the raw data used to calculate the agreement between both methods at the dichotomous level ‘mutation detected’ versus ‘no mutation detected’. The overall agreement for 182 samples was 96.7% with a lower limit of the 95% confidence interval (CI) of 93.0%. Positive agreement was calculated to be 98.8% with a lower limit of the 95% CI of 93.2%, while the negative agreement was calculated to be 95.1% with a lower limit of the 95% CI to be 89.0%. Finally, the KAPPA statistic of 93.3% indicated almost perfect agreement.

**Table 2:** Agreement table at the dichotomous level for valid, non-missing results

mCRC samples (*n* = 182)	*therascreen* KRAS RGQ PCR Kit		
Idylla™ KRAS Mutation Test	KRAS mutation	KRAS Wild-type	Totals
KRAS Mutation	79	5	84
KRAS Wild-type	1	97	98
Totals	80	102	182

Overall agreement between *therascreen* and Idylla™ platform for detection of KRAS mutations. *mCRC* metastatic colorectal cancer. KRAS Wild type = no mutation detected

### Comparison between *therascreen* and Idylla™ results at the mutation-specific level

The details for the different mutation-specific types as detected by Idylla™ and *therascreen* at the mutation-specific level are shown in Table 3.

**Table 3:** Agreement table at the mutation specific level

Idylla™	G12A	G12C	G12D	G12R	G12S	G12V	G13D	No mutation detected	Invalid	*A59E/P/V*	*Q61H/H2*	*Q61K/K2*	*Q61L/R*	*K117N1/N2*	*A146T/V/0*	Total
therascreen																
12ALA	6							1^a^								7
12CYS		6												***1***		7
12ASP			25													25
12ARG				3												3
12SER					6	**1**	**1**									8
12VAL						15			1						***1***	17
13ASP							16									16
No mutation detected				1^a^		1^a^	3^a^	97		*1*	*3*			*2*	*4*	112
Invalid/Run Control failed		2	1		3	2	1	41	6						*1*	57
Total	6	8	26	4	9	19	21	139	7	*1*	*3*	*0*	*0*	*3*	*6*	252

Genotyping concordance between the Idylla™ test and the reference test. Italic: discordant by design specimens. ^a^ True discordant samples. Bold: mutant-mutant discordant results

From this Table 3, it is clear that two mutant-mutant discordant results were detected, meaning that both methods detected a mutation, but the mutation was differently genotyped: two samples were diagnosed with a G12V and G13D mutation using Idylla, but *therascreen* detected this in both cases as a 12SER mutation. Furthermore, 13 samples contained a mutation that could not be detected by the *therascreen* assay since they were present in exon 3 and 4. These samples are therefore discordant-by-design specimens, and were subsequently excluded from agreement calculations. Within these 13 samples, two mutant-mutant discordants were found that are however concordant in respect to the dichotomous result ‘mutation detected’ or ‘no mutation detected’ (italic and bold in Table 3).

### Discordant testing

Overall, 6 true discordant results were noted, indicated in with an asterisk (*) in Table 3 and summarized in Table 4.

**Table 4:** True discordant results at the dichotomous level “mutation detected” versus “no mutation detected” for mutations in exon 2

Sample ID	Idylla™	*therascreen*	Confirmatory testing	New DNA extraction?
S8251	No mutation detected	12ALA	No mutation detected	Yes
S8261	G13D	No mutation detected	No mutation detected	Yes
S8320	G12R	No mutation detected	No mutation detected	No
S8322	G12V	No mutation detected	G12V	Yes
S8382	G13D	No mutation detected	No mutation detected	Yes
S8505	G13D	No mutation detected	No mutation detected	Yes

Listing of the samples with a true discordant result, namely mutated versus not mutated *KRAS*. Confirmatory testing of mutational status of the samples are shown in column 3

One mutation detected by the comparator method was missed by Idylla™, and 5 mutations detected by Idylla™ were not detected by the comparator method. These discordant samples were further investigated. The method of choice to perform the analysis of these discordant samples was the SOMATIC 1 KRAS/NRAS/BRAF kit (Multiplicom). This kit allows parallel detection of mutations in all exons of *KRAS*, *NRAS* and exon 15 of *BRAF* using massive parallel sequencing (MPS). MPS was chosen since it allows an analysis at low variant allele frequency (VAF), comparable to what can be achieved with Idylla™ and *therascreen*. It was preferred to use freshly isolated genomic DNA starting from FFPE slides, but due to limitations of the available material, a new extraction could only be done for 5 out of the 6 samples (Table 4). However, massive parallel sequencing was performed for all 6 samples. A first step with the chosen MPS method requires a Quality Control check of the extracted DNA. Only One (1) sample fulfilled the criteria, namely Sample S8322. Targeted sequencing confirmed the G12V mutation as detected with the Idylla™ platform. The VAF of this mutation, which weakly correlates with the tumor content in the sample, was 37%. MPS was not successful in confirmatory testing in five out of six cases. For these cases, conventional Sanger sequencing was used as the method of choice. For all five cases, the result obtained with the comparator method could be confirmed. All five samples had a tumor tissue content of over 15%, suggesting that results were not hampered by a lack of analytical sensitivity of this approach.

### Confirmatory testing of exon 3 or 4 Idylla results

Whenever one of the 14 mutations in *KRAS* exon 3 or 4 (A59E, A59G, A59T, Q61K, Q61K, Q61L, Q61R, Q61H, Q61H, K117N, K117N, A146P, A146T, A146V) was detected by the Idylla™ KRAS Mutation Test, they could not be verified by design with the *therascreen* KRAS RGQ kit. Although the 13 samples, showing one of these mutations (Table 3: italic), were not taken into account for agreement calculations, it was considered interesting to confirm the presence of the mutation by investigating them with routine reference approaches, namely MPS or Sanger Sequencing. Furthermore, the 2 mutant-mutant discordants (Samples S8494 and S8546 in Table 5) were investigated as well. The results of these 15 samples are summarized in Table 5.

**Table 5:** Confirmatory testing using routine reference approaches

Sample ID	Idylla™	Therascreen	Confirmatory result	Reference approach	VAF (%)
S8470	A146P/T/V	N/A	A146T	MPS	31
S8476	A146P/T/V	N/A	A146T	MPS	20
S8342	A146P/T/V	N/A	A146V	MPS	2
S9195	A146P/T/V	N/A	A146T	MPS	30
S8426	A146P/T/V	12VAL	A146V	Sanger	N/A
S8383	A146P/T/V	N/A	A146T	Sanger	N/A
S8397	K117N	12CYS	K117N	Sanger	N/A
S8250	K117N	N/A	Q61K	MPS	23
S8475	K117N	N/A	K117N	MPS	70
S8431	A59T/E/G	N/A	No mutation detected	MPS	N/A
S8233	Q61H	N/A	Q61H	Sanger	N/A
S8377	Q61H	N/A	Q61H	Sanger	N/A
S8956	Q61H	N/A	Q61H	MPS	42
S8494	G12V	12SER	G12V	MPS	37
S8546	G13D	12SER	G12C	MPS	33

Confirmatory testing of Idylla results using massive parallel sequencing (MPS) or Sanger Sequencing. Samples 1–13 were discordant-by-design mutations and Samples 14–15 were mutant-mutant discordants. Samples 1–13 were not taken into account for agreement calculations

From Table 5, one can understand that out of the 13 samples that yielded as successful result, 11 samples had a mutation specific concordant result to the Idylla™ result. For 3 samples (S8250, S8431, S8546), a discordant result was obtained between the Idylla™ KRAS Mutation Test and the reference approach: one sample (S8431) was identified as *KRAS* wild-type and two samples (S8250 and S8546) were discordant at the mutation-specific level, but concordant with Idylla™ at the dichotomous ‘mutation detected’ level.

## Discussion

Testing for presence of hotspot mutations in exons 2, 3 and 4 of the *KRAS* gene has become standard of care in the mCRC setting [3]. Several clinical trials indicate that patients with tumors wild-type for *KRAS* benefit from anti-EGFR antibody therapy [20]. Two methods are currently FDA approved and could therefore be considered as the current gold standard for testing in FFPE material, namely the *therascreen* KRAS RGQ PCR Kit (Qiagen) and the cobas® KRAS Mutation Test (Roche) [17]. These methods are however hampered by the fact that they only detect and identify mutations in exon 2 or that they do not discriminate between mutations at the amino acid level for exon 2 or 3, respectively. In addition, both methods require pooling of samples for optimal kit usage and require tissue deparaffination, DNA quantitation and manual isolation of genomic DNA [17]. This retrospective study compared the *therascreen* RGQ KRAS Mutation Kit to the Idylla™ KRAS Mutation Test. This latter platform allows detection of 21 *KRAS* mutations in hotspot amino acids of exon 2, 3 and 4 using simultaneous DNA extraction and qPCR reaction in a single-use cartridge, directly on FFPE, with a result within 2 h.

Several parameters were evaluated, such as the percentage necrotic tissue in relation to the number of valid Idylla™ KRAS results, as well as small tissue area and the overdue age of the samples, defined as the number of days between sectioning and testing. For these 3 parameters no correlation could be found, indicating that the Idylla™ KRAS Mutation test is a robust assay, overcoming less optimal sample conditions (data not shown). Moreover, out of the 31 samples that did not reach the cut-off of 50 mm^2^, a valid result could be obtained in 30 cases. This result suggests that the test might be more sensitive than stated for small tumor areas. We further investigated the impact of the sample collection date on the number of invalid results and a significant association was found between samples older than 4 years (*n* = 14) and the number of invalid results (*n* = 6) (p < 0.05). Pre-analytical conditions of the samples, such as time of fixation, were highly controlled in the lab, suggesting the invalid results are a result of the storage of the FFPE blocks influencing the DNA integrity and hence its amplification in PCR reactions as was previously demonstrated [21]. The impact of even older archived samples on KRAS mutation analysis could not be assessed due to ethical limitations imposed by the ethical committee. The clinical importance of these findings is however expected to be of lesser importance, since KRAS biomarker determination is most likely to be requested within 4–5 years after sample collection.

Comparison of the Idylla™ platform with the *therascreen* assay was performed for 252 samples, generating 194 valid results overall with both methods. There were respectively 57 and 7 invalid or failed results with *therascreen* and Idylla™, respectively. Of note, the samples that turned out to be invalid with the Idylla™ platform were all but one also invalid using *therascreen*. The success rate of the Idylla™ platform is therefore 97.2% and is hence considered acceptable within the scope of this study. Of note, the success rate of *therascreen* was only a disappointing 77.4% due to invalid calls (*n* = 33) and test failures after QC (*n* = 24). It is difficult to explain this lower success rate, but a possible explanation might lie in tissue loss during the DNA extraction process. Since manual DNA extraction is a separate step in the DNA extraction process, it seems possible that tissue got lost, eg. during tissue scraping/collection or column purification, a step which is not required with Idylla™. Of note, 16 samples had a tissue surface area of less than 25 mm^2^ on HE, all of which were invalid after QC step using *therascreen*. One can imagine that even a small loss of tissue might hamper the subsequent DNA steps. No further explanation could be found, not in the number of used slides nor in the technician performing the test or the age of the samples. Such performance would hamper the laboratory workflow efficiency.

Since the diagnostic yield of the Idylla™ platform is higher compared to *therascreen* due to the inclusion of exon 3 and 4 mutations, agreement at the dichotomous level ‘mutation detected’ versus ‘mutation not detected’ could only be performed for 182 out of 194 results. The overall agreement between both methods was 96.7% [95% CI: 93.0%-98.5%], with corrected kappa statistics of 0.93 (Table 2), indicating that both methods almost perfectly agree. For 6 samples, agreement was not obtained and further investigation was warranted. First, in order to avoid any bias, we performed a new genomic DNA isolation whenever sufficient material was available. Second, using MPS or Sanger sequencing we could confirm for two samples the result as detected by the Idylla™ platform and for four samples the results as obtained with *therascreen* (Table 4). Of note, the confirmation of the wild-type status of sample S8251 was only possible after a new DNA extraction, pointing out a possible sample contamination. One could suggest that the reason for the discordant results might be the different limit of detection of both tests, although both the *therascreen* KRAS RGQ PCR Kit and the CE-IVD Idylla™ KRAS Mutation Test claim to detect mutations down to the 5% limit of detection. The LOD of the Idylla™ KRAS Mutation Test was briefly verified and we were able to detect previously determined *KRAS* mutations in 4 clinical samples with a TTC content of 5-10% (Table 1). Of note, the detection limit of G13D mutations was published by the companies in the kit inserts to be slightly lower for the *therascreen* method as compared to the Idylla™ KRAS Mutation test (6.25% versus 5%), providing a possible explanation for the difference in detection of this mutation in three out of four samples. However, analysis with Sanger sequencing as a reference approach revealed that no *KRAS* mutation was present. Since these 4 samples contained a TTC of 10% or higher, we believe that these wild-type results are not false-negative results, but it cannot be completely excluded. Re-analysis with the Idylla™ platform could not be performed due to insufficient DNA material.

As mentioned before, the diagnostic yield of the Idylla™ platform is higher due to the inclusion of mutation analysis of hotspot amino acids in exon 3 and 4. Overall, the Idylla™ platform identified 13 samples with a mutation in exon 3 or 4, meaning that 12% (*n* = 13/106) of the mutated samples would not have been detected using the *therascreen* reference test, a percentage in accordance with previous results [13]. In addition, two samples, concordant at the mutation-mutation level, but discordant for the type-specific mutation, were further investigated MPS. Several studies suggested that the type of mutation might be of importance in predicting the response to anti-EGFR therapy or to other future therapies and that it might not be sufficient to limit results to ‘mutation (not) detected’ [13, 22]. In addition, it is advisable to determine the specific mutation in tissue samples whenever these results would be used in patient management to non-invasively monitor responses to treatment [20]. It therefore seems to be useful at this point to carefully determine the mutation down to the amino acid level. In view of these considerations, we not only searched further confirmation for the 13 mutations in *KRAS* exon 3 or exon 4, but also for the two exon 2 discordant mutations at the amino acid level. (Table 5).

The Idylla™ platform identified correctly the *KRAS* mutational status as compared to a reference approach in 10 out of the 13 valid cases (Table 5). When inspecting further the three discordant results (S8250, S8431 and S8546), a possible explanation might be a sample switch or a false-positive call by the Idylla instrument. We therefore performed additional tests and verified with a new FFPE slide for sample S8250 the Idylla™ KRAS Mutation Test result. Surprisingly, we now detected the Q61K mutation as was confirmed with MPS and not the K117N mutation as before. This could mean either a sample switch or sample contamination during the initial Idylla™ KRAS test or alternatively, the presence of two mutations in the sample with almost equal Ct. In this latter case, the Idylla™ platform would call only one mutation, depending on the ΔCq value. However, the K117N mutation could not be detected with MPS or Sanger, meaning that a sample switch or a true false-positive call by the instrument are the most probable reasons. The other two discordant samples could not be verified with the Idylla™ KRAS Mutation Test anymore due to lack of available FFPE material. However, a sample switch is probably not the reason for the discordant result for S8431, since no other sample in this study was positive for this *KRAS* A59T/E/G mutation. This probably represents therefore true false positive result. The third discordant sample (S8546) generated three different mutational calls with three different methods, suggesting that this sample should be overall repeated or excluded from analyses.

Overall, out of the 195 valid results obtained with the Idylla™ KRAS Mutation Test, the concordance with *therascreen* or another reference approach was calculated as 96.9% (*n* = 189/195). Overall, regardless of the confirmatory testing, one could conclude that the overall agreement between the Idylla™ KRAS Mutation Test and the *therascreen* RGQ KRAS PCR Kit is very good, namely 96.7% [95%CI: 93.0%-98.5%] and that Idylla has the superior quality of detecting hotspot mutations in exons 3 and 4 whilst generating fewer invalid results.

The current landscape of mutation analysis is quickly changing due to large exome and genome sequencing projects [23]. This will possibly lead to the detection of very rare mutations in *KRAS* that might be clinically relevant, but will probably occur with a frequency that is too low to ever firmly state their clinical relevance. The Idylla™ platform counterbalances this by offering a quick, on-demand system to screen for hotspot mutations with well-known clinical relevance. On the other hand, this is also the drawback of any available PCR based method, since emerging mutations will not be detected with any current KRAS Mutation PCR Test. In addition, for high-throughput laboratories, it might be more cost-effective to screen mCRC samples with MPS to have more output at once, but at a slower pace. It should also be noted that testing for *NRAS* mutations in *KRAS* wild-type tumors is now standard of care [3]. Since almost 50% of the tested samples will return a wild-type *KRAS* result, these samples should be tested for *NRAS* mutations [10, 11]. Therefore, it would be valuable in terms of cost, labor and lab workflow efficiency to have an extended RAS Test that included an ‘*NRAS* mutation detected’ versus ‘*NRAS* mutation not detected’ result together with the KRAS result generated with the same RAS cartridge tested. Meanwhile a separate Idylla™ NRAS/BRAF Mutation Test for CRC with a similar turnaround time of approximately 2 h became available which could address this unmet clinical need.

## Conclusions

Overall, the Idylla™ system was able to correctly identify the *KRAS* status in a high number of clinical FFPE tissue samples, whilst being highly automated and allowing for a rapid outcome which is needed to adequately guide therapy in mCRC patients.

## Abbreviations

12ALA: p.Gly12Ala; c.35G > C
12ARG: p.Gly12Arg; c.34G > C
12ASP: p.Gly12Asp; c.35G > A
12CYS: p. Gly12Cys; c.34G > T
12SER: p.Gly12Ser; c.34G > A
12VAL: p. Gly12Val; c.35G > T
13ASP: p.Gly13Asp; c.38G > A
A146P: p.Ala146Pro; c.436G > C
A146T: p.Ala146Thr; c.436G > A
A146V: p.Ala146Val; c.437C > T
A59E: p.Ala59Glu; c.176C > A
A59G: p.Ala59Gly; c.176C > G
A59T: p.Ala59Thr; c.175G > A
CI: Confidence interval
CRC: Colorectal cancer
FFPE: Formalin fixed paraffin embedded
G12A: p.Gly12Ala; c.35G > C
G12C: p. Gly12Cys; c.34G > T
G12D: p.Gly12Asp; c.35G > A
G12R: p.Gly12Arg; c.34G > C
G12S: p.Gly12Ser; c.34G > A
G12V: p. Gly12Val; c.35G > T
G13D: p.Gly13Asp; c.38G > A
K117N: p.Lys117Asn; c.351A > C
K117N: p.Lys117Asn; c.351A > T
LOD: Limit of detection
mCRC: metastatic CRC
MPS: Massive parallel sequencing
Q61H: p.Gln61His; c.183A > C
Q61H: p.Gln61His; c.183A > T
Q61K: p.Gln61Lys; c.180_181delinsAA
Q61K: p.Gln61Lys; c.181C > A
Q61L: p.Gln61Leu; c.182A > T
Q61R: p.Gln61Arg; c.182A > G
VAF: Variant allele frequency

## References

[1] Kuipers EJ, Grady WM, Lieberman D, Seufferlein T, Sung JJ, Boelens PG, van de Velde CJ, Watanabe T (2015). Colorectal Cancer. Nat Rev Dis Primers.

[2] Siegel RL, Miller KD, Jemal A (2015). Cancer statistics, 2015. CA Cancer J Clin.

[3] Van Cutsem E, Cervantes A, Nordlinger B, Arnold D (2014). Metastatic colorectal cancer: ESMO Clinical Practice Guidelines for diagnosis, treatment and follow-up. Ann Oncol.

[4] Ciardiello F, Tortora G (2008). EGFR antagonists in cancer treatment. N Engl J Med.

[5] Moorcraft SY, Smyth EC, Cunningham D (2013). The role of personalized medicine in metastatic colorectal cancer: an evolving landscape. Ther Adv Gastroenterol.

[6] Douillard JY, Oliner KS, Siena S, Tabernero J, Burkes R, Barugel M, Humblet Y, Bodoky G, Cunningham D, Jassem J (2013). Panitumumab-FOLFOX4 treatment and RAS mutations in colorectal cancer. N Engl J Med.

[7] Van Cutsem E, Kohne CH, Hitre E, Zaluski J, Chang Chien CR, Makhson A, D'Haens G, Pinter T, Lim R, Bodoky G (2009). Cetuximab and chemotherapy as initial treatment for metastatic colorectal cancer. N Engl J Med.

[8] Amado RG, Wolf M, Peeters M, Van Cutsem E, Siena S, Freeman DJ, Juan T, Sikorski R, Suggs S, Radinsky R (2008). Wild-type KRAS is required for panitumumab efficacy in patients with metastatic colorectal cancer. J Clin Oncol.

[9] De Roock W, Claes B, Bernasconi D, De Schutter J, Biesmans B, Fountzilas G, Kalogeras KT, Kotoula V, Papamichael D, Laurent-Puig P (2010). Effects of KRAS, BRAF, NRAS, and PIK3CA mutations on the efficacy of cetuximab plus chemotherapy in chemotherapy-refractory metastatic colorectal cancer: a retrospective consortium analysis. Lancet Oncol.

[10] NCCN. NCCN Guidelines Version 2.2016 Colon Cancer. In*.*; 2016.

[11] NCCN. NCCN Guidelines Version 2.2016 Rectal Cancer. In*.*; 2016.

[12] Bardelli A, Siena S (2010). Molecular mechanisms of resistance to cetuximab and panitumumab in colorectal cancer. J Clin Oncol.

[13] Peeters M, Kafatos G, Taylor A, Gastanaga VM, Oliner KS, Hechmati G, Terwey JH, van Krieken JH (2015). Prevalence of RAS mutations and individual variation patterns among patients with metastatic colorectal cancer: A pooled analysis of randomised controlled trials. Eur J Cancer.

[14] Ogino S, Kawasaki T, Brahmandam M, Yan L, Cantor M, Namgyal C, Mino-Kenudson M, Lauwers GY, Loda M, Fuchs CS (2005). Sensitive sequencing method for KRAS mutation detection by Pyrosequencing. J Mol Diagn.

[15] Whitehall V, Tran K, Umapathy A, Grieu F, Hewitt C, Evans TJ, Ismail T, Li WQ, Collins P, Ravetto P (2009). A multicenter blinded study to evaluate KRAS mutation testing methodologies in the clinical setting. J Mol Diagn.

[16] Tsiatis AC, Norris-Kirby A, Rich RG, Hafez MJ, Gocke CD, Eshleman JR, Murphy KM (2010). Comparison of Sanger sequencing, pyrosequencing, and melting curve analysis for the detection of KRAS mutations: diagnostic and clinical implications. J Mol Diagn.

[17] Angulo B, Lopez-Rios F, Gonzalez D (2014). A new generation of companion diagnostics: cobas BRAF, KRAS and EGFR mutation detection tests. Expert Rev Mol Diagn.

[18] Newcombe RG (1998). Interval estimation for the difference between independent proportions: comparison of eleven methods. Stat Med.

[19] Altman (1991). Practical Statistics for medical research.

[20] Ray K (2015). Colorectal cancer: Liquid biopsy enables real-time monitoring of molecular alterations in CRC. Nat Rev Gastroenterol Hepatol.

[21] Adema V, Torres E, Sole F, Serrano S, Bellosillo B (2014). Paraffin treasures: do they last forever?. Biopreservation Biobanking.

[22] Tejpar S, Celik I, Schlichting M, Sartorius U, Bokemeyer C, Van Cutsem E (2012). Association of KRAS G13D tumor mutations with outcome in patients with metastatic colorectal cancer treated with first-line chemotherapy with or without cetuximab. J Clin Oncol.

[23] Network CGA (2012). Comprehensive molecular characterization of human colon and rectal cancer. Nature.

